# Polymorphisms in the *SOCS7* gene and glucose homeostasis traits

**DOI:** 10.1186/1756-0500-6-235

**Published:** 2013-06-15

**Authors:** Melissa M Capuano, John D Sorkin, Yen-Pei C Chang, Hua Ling, Jeffrey R O’Connell, Paul B Rothman, Braxton D Mitchell, Kristi D Silver

**Affiliations:** 1 Division of Endocrinology, Diabetes, and Nutrition, University of Maryland School of Medicine, 660 West Redwood Street, Room 494, Baltimore, MD 21201, USA; 2 Division of Gerontology, University of Maryland School of Medicine, Baltimore, MD USA; 3 Division of Geriatrics and Gerontology, Department of Medicine, Department of Veterans Affairs, Veterans Affairs Medical Center, Baltimore, MD USA; 4 Baltimore Geriatric Research, Education and Clinical Center, Baltimore, MD USA; 5 Department of Internal Medicine, University of Iowa, Iowa City, IA, USA

**Keywords:** Type 2 diabetes, Genetics, *SOCS7* gene, Polymorphism

## Abstract

**Background:**

SOCS7 is a member of the suppressor of cytokine signaling family of proteins and is expressed in skeletal muscle and islets. *SOCS7* deficient mice develop islet hyperplasia in the setting of increased insulin sensitivity and normal glucose tolerance. The objective of this study was to determine if variants in *SOCS7* play a role in variation of glucose and insulin levels and the development of type 2 diabetes (T2DM).

**Results:**

Five *SOCS7* tagging SNPs were genotyped in diabetic and nondiabetic Old Order Amish. A case–control study was performed in T2DM (n = 145) and normal glucose tolerant (n = 358) subjects. Nominal associations were observed with T2DM and the minor alleles for rs8068600 (P = 0.01) and rs8074124 (P = 0.04); however, only rs8068600 remained significant after Bonferroni adjustment for multiple comparisons (P = 0.01). Among nondiabetic Amish (n = 765), no significant associations with glucose or insulin traits including fasting or 2 hour glucose and insulin from the oral glucose tolerance test, insulin or glucose area under the curve, Matsuda Index or HOMA-IR were found for any of the SNPs.

**Conclusion:**

In conclusion, genetic variants in the *SOCS7* gene do not impact variation in glucose homeostasis traits and only minimally impact risk of T2DM in the Old Order Amish. Our study was not able to address whether rare variants that potentially impact gene function might influence T2DM risk.

## Background

The suppressor of cytokine signaling (SOCS) proteins are a family of eight proteins that negatively regulate cytokine signaling pathways via inhibition of JAK/STAT signal transduction [1-3]. Expression of SOCS proteins is induced by proinflammatory cytokines including IL1-beta, INF-gamma, TNF-alpha, IL-6 and growth hormone [4-7]. In animal models, SOCS proteins 1 and 3 have been shown to play a role in insulin signaling and diabetes [7-14]; however, less is known about other members of the SOCS family and their role in the development of diabetes. In C57BL/6J mice, *SOCS7* is expressed at highest levels in skeletal muscle, pancreatic islets, and brain [15]. *SOCS7* deficient mice develop islet hyperplasia [15,16] and on a mixed C57BL/6J and 129S6/SvEvTac background also experience increased insulin sensitivity as demonstrated by lower glucose levels and prolonged hypoglycemia during an insulin tolerance test, and increased glucose clearance during an intraperitoneal glucose tolerance test [15]. These data suggest that *SOCS7* plays a role in regulating glucose homeostasis. A proposed molecular mechanism for this regulation is through *SOCS7* targeting of insulin receptor substrate (IRS) proteins for ubiquitination and proteasomal degradation. This action would decrease insulin signaling, and thereby, increase insulin resistance. Decreased insulin signaling via decreased IRS levels has also been proposed as the mechanism whereby *SOCS7* affects islet size [15].

The *SOCS7* gene contains 10 exons over a 45 kb region on human chromosome 17q12. In humans, *SOCS7* is expressed diffusely with high levels in testis, ovaries, spleen, brain and spinal cord and moderate levels in pancreatic islets (http://www.genecards.org). As a result of *SOCS7*’s role in glucose homeostasis in mice, we hypothesized that variants in *SOCS7* might contribute to variation in glucose and insulin levels as well as the development of Type 2 diabetes (T2DM) in humans. To test this hypothesis, we genotyped a panel of tagging SNPs in the *SOCS7* gene in the Old Order Amish of Lancaster, Pennsylvania and assessed associations with T2DM and insulin and glucose related traits.

## Methods

### Old Order Amish of Lancaster Pennsylvania

The Old Order Amish are a genetically well-defined Caucasian founder population. Nearly all of these individuals share common ancestors insofar as the entire Amish community of Lancaster County (now numbering over 30,000 individuals) can be connected into a single 14-generation pedigree [17].

The Amish Family Diabetes Study (AFDS) was initiated in 1995 with the goal of identifying susceptibility genes for T2DM and related traits. A description of subject recruitment and the examination protocol has been previously reported [18]. Briefly, probands with T2DM and their extended family members were invited to participate in the study. After an overnight fast, a 75 gram oral glucose tolerance test (OGTT) was administered to all subjects without a prior history of diabetes. Blood samples were sent for analysis of glucose and insulin concentrations. Glucose concentrations were assayed with a Beckman glucose analyzer (Beckman Coulter, Fullerton, CA) using the glucose oxidase method (interassay coefficient of variation = 1.52%). Insulin levels were determined by radioimmunoassay (Linco, St. Louis, MO) (interassay coefficient of variation = 4.42%).

Criteria for the diagnosis of diabetes were adapted from American Diabetes Association 1997 recommendations [19]. Diabetes status was defined by a single fasting venous plasma glucose level ≥7 mmol/l, a 2 hour OGTT venous plasma glucose level ≥11.1 mmol/l, current treatment with insulin and/or oral hypoglycemic agents, or by confirmed diagnosis by a physician. Impaired glucose tolerance (IGT) status was defined as a 2 hour OGTT glucose value between 7.8 and 11.1 mmol/L. Normal glucose tolerance (NGT) was defined as having a fasting glucose <6.1 mmol/L and a 2 hour OGTT glucose <7.8 mmol/L. Diabetic subjects with an age at diagnosis <35 years were reclassified as diabetes status unknown in order to minimize heterogeneity due to inclusion of subjects with type 1 diabetes. For the case–control study, a total of 503 Amish subjects [T2DM (n = 145), and NGT (n = 358) aged 38 years or older] from the Amish Family Diabetes Study (AFDS) were included in the analysis (Table 1).

**Table 1:** Clinical characteristics of the Amish*

**Trait**	**T2DM**	**NGT**	**NGT + IGT**
	**(n = 145)**	**(n = 358)**	**(n = 765)**
% female	69.7	48.0	52.6
Age (yrs)	65 ± 12	51 ± 11	44.3 ± 14.7
BMI (kg/m^2^)	30.1 ± 4.0	27.4 ± 4.7	27.0 ± 4.8
% IGT	-	-	19.1
Age of onset of DM (yrs)	59 ± 11	-	-
HOMA-IR	-	2.5 ± 1.5	2.5 ± 1.4
Fasting Glucose (mmol/L)	-	5.04 ± 0.42	5.02 ± 0.46
2 hour Glucose (mmol/L)	-	5.76 ± 1.20	6.21 ± 1.7
Fasting Insulin^†^ (pmol/L)	-	68.1 (55.6, 86.8)	66.7 (54.9, 86.1)
2 hour Insulin^†^ (pmol/L)	-	211.8 (126.4, 341.0)	218.8 (128.5, 364.6)
AUC Glucose (mmol/L)	-	349.2 ± 51.5	359.9 ± 67.2
AUC Insulin^†^ (pmol/L)	-	761.9 (540.3, 1046.6)	752.1 (525.0, 1046.6)

* mean ± SD unless otherwise indicated.

^†^median (25th, 75th percentile).

Association studies with glucose homeostasis measures including fasting glucose, fasting insulin, 2 hour glucose, 2 hour insulin, area under the curve (AUC) glucose, AUC insulin, HOMA-IR and Matsuda Index were performed in a larger sample of 765 nondiabetic Amish subjects that included the 358 NGT subjects described above, an additional 218 NGT subjects, and 148 IGT subjects. Area under the curve was calculated using the trapezoid rule. Homeostasis model assessment of insulin resistance (HOMA-IR) was calculated according to the following formula: HOMA-IR = [fasting serum insulin (mU/L) x fasting plasma glucose (mmol/L)]/22.5 [20]. The Matsuda Index was calculated to assess insulin resistance [21].

All study protocols were approved by the Institutional Review Board at the University of Maryland School of Medicine. Informed consent was obtained from each study participant prior to enrollment.

### Genotyping

**Figure 1:**
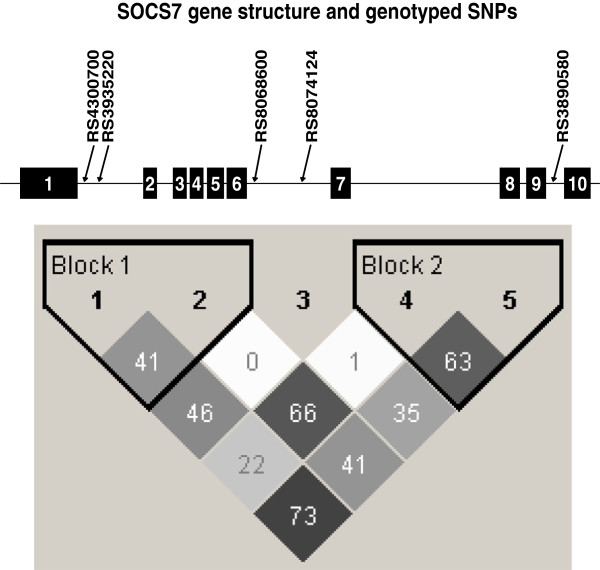
***SOCS7 *gene structure and genotyped SNPs.** The 5 SNPs are represented by the numbers 1–5 above the correlation matrix. The numbers within each box of the correlation matrix represent pairwise r^2^ value between SNPs.

DNA was extracted from leukocytes for genotyping. We selected 5 SNPs (from a total of 36 SNPs represented in HapMap CEU) that tagged *SOCS7* and the 5 kb region upstream and downstream of the gene. These SNPs captured 100% of common variants (>5%) in HapMap CEU in the *SOCS7* gene at r^2^ > 0.95 (HapMap Release 36). Figure 1 shows the locations of these SNPs relative to the 10 exons and their LD structure. Rs3890580 was genotyped by pyrosequencing (PSQ HS 96MA System; Pyrosequencing AB, Uppsala, Sweden) according to the manufacturer’s protocol. Rs4300700 was genotyped using either TaqMan SNP Genotyping Assays (Applied Biosystems; Foster City, CA) according to the manufacturer’s protocol or by dideoxy sequencing on an ABI 3730xl sequencer. Rs3935220, rs8068600 and rs8074124 were genotyped by either pyrosequencing or TaqMan. At least 5% duplicate samples were included to determine genotype concordance rates within the same platform and between different genotyping platforms, which were 95-100%. Discordant samples were excluded from analysis.

Genome wide association study (GWAS) *SOCS7* SNP association data was downloaded from the Meta-Analyses of Glucose and Insulin-related traits Consortium (MAGIC) web site (http://www.magicinvestigators.org/downloads/) for HOMA-IR, fasting insulin, fasting glucose and 2 hour glucose [22,23]. The National Human Genome Research Institute (NHGRI) database (http://www.genome.gov/gwastudies) was used to search for *SOCS7* SNPs associated with T2DM as well as glucose and insulin related traits [24].

### Statistical analysis

Before analysis, Amish genotypes were checked for Mendelian consistency using the PedCheck software program [25] in the extended Amish pedigree. A small number of Mendelian errors (<1.5% of genotypes) were either resolved or removed before analysis. Observed genotypes for all SNPs were tested for fit with those expected under Hardy-Weinberg equilibrium using the Chi square test; all genotype distributions conformed to Hardy-Weinberg expectations.

Quantitative trait means were estimated according to *SOCS7* genotypes in the 765 nondiabetic Amish subjects. To account for the relatedness among family members, the measured genotype approach was used [26] in which we estimated the likelihood of specific genetic models given the pedigree structure. Parameter estimates were obtained by maximum likelihood methods, and the significance of association was tested by likelihood ratio tests. Analyses were performed using recessive, dominant and additive models. Within each model, we simultaneously estimated the effects of age and sex, and age, sex and body mass index (BMI). Quantitative trait analyses were conducted using the SOLAR software program [27]. To account for the multiple SNPs tested, we considered a P-value of 0.01 to be statistically significant. Analysis of the diabetes trait was made using the dichotomous trait extension to the SOLAR software program, which employs a threshold model to assess the effect of genotype on the binary outcome.

## Results

Characteristics of the Amish subjects are presented in Table 1. To determine if SNPs in the *SOC7* gene contribute to the development of T2DM, we employed a case–control analysis utilizing 145 T2DM cases and 358 NGT controls from the AFDS. Subjects with T2DM were older, had a higher BMI and were more likely to be female. Nominal associations with T2DM were found for the minor allele for rs8068600 (P = 0.01) and rs8074124 (P = 0.04) (Table 2, Additional file 1: Table S1); however, after adjustment for multiple comparisons, only rs8068600 met the Bonferroni-adjusted threshold for statistical significance (P = 0.01). No significant or nominally significant (P < 0.05) associations with glucose or insulin traits including fasting or 2 hour glucose and insulin from the OGTT, Insulin AUC, Glucose AUC, Matsuda Index or HOMA-IR were found for any of the SNPs in the Amish after adjustment for age and sex. Similar results were found when BMI was included in the model. Data from the MAGIC GWAS identified a nominal association of rs4300700 with ln fasting insulin (P = 0.01). No additional associations were found in the MAGIC data for *SOCS7* tagging SNPs for ln fasting insulin, fasting glucose, 2 hour glucose or HOMA-IR [22,23]. Review of the NHGRI GWAS database did not reveal any *SOCS7* SNP associations with T2DM or other glucose homeostasis traits; however, SNPs included in the database are limited to those with P-values <1.0 × 10^-5^[24].

**Table 2:** Allele frequencies and results of association analysis

**SNP name**	**Major/minor allele**	**Minor allele frequency**	**Matsuda index**		**Fasting glucose**		**Diabetes vs. control**	
			**P value**	**β coefficient**	**P value**	**β coefficient**	**P value**	**Odds ratio**
rs4300700	G/A	0.08	0.98	−0.006	0.96	0.043	0.84	0.96
rs3935220	G/C	0.09	0.31	0.227	0.26	−0.887	0.99	1.00
rs8068600	G/C	0.17	0.25	0.236	0.98	−0.021	0.01	1.59
rs8074124	T/C	0.22	0.46	0.133	0.40	−0.524	0.04	1.39
rs3890580	T/A	0.20	0.45	0.144	0.19	−0.864	0.15	1.27

Linkage disequilibrium (LD) analysis in the Amish revealed some LD among most of the *SOCS7* SNPs (r^2^ = 0-0.73; see Figure 1). The closest genes 5′ of *SOCS7* are *GPR179* (8.3 kb) and *MRPL45* (28.9 kb) and the closest gene 3′ is *SNIP* (178.3 kb). None of these genes are in the same LD block as the *SOCS7* gene.

## Discussion and conclusion

Based on studies in mice and other members of the SOCS family of proteins, *SOCS7* is an excellent candidate gene for regulation of glucose homeostasis traits and T2DM as a mutation in the *SOCS7* gene causing decreased or absent function of the SOCS7 protein would be expected to improve insulin sensitivity and decrease the risk of T2DM while an activating mutation in the *SOCS7* gene would be expected to increase insulin resistance and the risk of T2DM [12,14,15,28,29]. Despite genotyping SNPs in and adjacent to the *SOCS7* gene, SNP genotype did not significantly impact risk of T2DM, measures of insulin resistance or glucose levels after adjustment for multiple comparisons. These results are consistent with findings in MAGIC and other GWAS [22-24]. Only one other candidate gene study has examined the role of genetic variation in the *SOCS7* gene with measures of insulin resistance. In contrast to our study, Tellechea et al. found an association between rs8074124 and HOMA-IR (P = 0.018) in 780 men with European ancestry seen at the University of Buenos Aires [30]. Differences in results may be due to the inclusion of women in our study as well as differences in recruitment strategies as the University of Buenos Aires population was recruited from the Department of Haemotherapy.

While the number of subjects included in our study was relatively small compared to those found in genome wide association studies, our sample of 765 subjects did none-the-less provide 80% power to detect an association that accounted for as little as 1.5% of the total variance in glucose and insulin homeostasis traits. One limitation of our study is that we were underpowered to detect modest effect sizes for T2DM in the case–control study as our sample provided 80% power to detect odds ratios of 1.6 to 1.8. While it is possible that *SOCS7* plays a role in T2DM and related glucose homeostasis traits in other populations, this scenario seems unlikely as many genetic findings in the Amish are similar to those found in the outbreed Caucasian population [31-33]. Furthermore, in *SOCS7* SNPs found on the DNA microarray chips used for T2DM and glucose homeostasis GWAS, no significant associations have been reported with these SNPs or other SNPs in the region of the *SOCS7* gene. Additionally, in studies by MAGIC, only one nominally associated SNP (rs4300700) was identified which is consistent with our findings in the Amish of a very limited to no impact of *SOCS7* on risk of T2DM or variation in glucose homeostasis traits [22]. Another limitation of our study is the higher number of female subjects in the T2DM group compared to the NGT and NGT + IGT groups. This difference may be due to selection bias related possibly to women being more health conscious and more willing to take advantage of the screening done as part of the study.

In summary, genetic variation in the *SOCS7* gene does not significantly impact glucose homeostasis traits or risk of T2DM in the Old Order Amish of Lancaster Pennsylvania.

## Abbreviations

AIRg: Acute insulin response to glucose; AUC: Area under the curve; AFDS: Amish Family Diabetes Study; BMI: Body mass index; GWAS: Genome wide association study; HOMA-IR: Homeostasis model assessment of insulin resistance; IFG: Impaired fasting glucose; IGT: Impaired glucose tolerance; IRS: Insulin receptor substrate; LD: Linkage disequilibrium; MAGIC: Meta-Analyses of Glucose and Insulin-related traits Consortium; NHGRI: National Human Genome Research Institute; NGT: Normal glucose tolerance; OGTT: Oral glucose tolerance test; SNP: Single nucleotide polymorphism; SOCS: Suppressor of cytokine signaling; T2DM: Type 2 diabetes.

## Competing interests

The authors have no competing interests to declare.

## Authors’ contributions

Conceived and designed the experiments: MMC. KDS, PBR; Performed the experiments: MMC, KDS; Analyzed the data: MMC, KDS, BDM, JRO, JDS. HL, YPCC; Wrote the paper: MMC, KDS, BDM; Reviewed Manuscript MMC, PBR, BDM, KDS, JDS. All authors read and approved the final manuscript.

## Supplementary Material

Additional file 1: Table S1 Genotype Frequency of *SOCS7* SNPS. Click here for file
