# Supplementary material for: Polymorphisms in the SOCS7 gene and glucose homeostasis traits
Source: BMC Res Notes. 2013 Jun 15;6:235. doi: 10.1186/1756-0500-6-235 (PMC3686602; doi:10.1186/1756-0500-6-235)
Supplement: Additional file 1: Table S1 — Genotype Frequency of SOCS7 SNPS. [file 1756-0500-6-235-S1.doc]

**Additional file 1 Table S1. Genotype Frequency of *SOCS7* SNPS**

| SNP | T2DM (n=145) | | | NGT (n=358) | | |
| --- | --- | --- | --- | --- | --- | --- |
|  | 11 | 12 | 22 | 11 | 12 | 22 |
| rs4300700 | 0.79 | 0.20 | 0.01 | 0.85 | 0.15 | 0.006 |
| rs3935220 | 0.81 | 0.18 | 0.01 | 0.83 | 0.16 | 0.008 |
| rs8068600 | 0.77 | 0.21 | 0.01 | 0.69 | 0.28 | 0.03 |
| rs8074124 | 0.67 | 0.30 | 0.03 | 0.61 | 0.34 | 0.05 |
| rs3890580 | 0.66 | 0.31 | 0.04 | 0.64 | 0.32 | 0.04 |
